# The Mega2R package: R tools for accessing and processing genetic data in common formats

**DOI:** 10.12688/f1000research.15949.2

**Published:** 2019-02-25

**Authors:** Robert V. Baron, Justin R. Stickel, Daniel E. Weeks

**Affiliations:** 1 Department of Human Genetics, Graduate School of Public Health, University of Pittsburgh, Pittsburgh, Pennsylvania, 15261, USA; 2 Department of Biostatistics, Graduate School of Public Health, University of Pittsburgh, Pittsburgh, Pennsylvania, 15261, USA

**Keywords:** database, genotypes, genome-wide association studies, linkage, Mega2, phenotypes, R, SQLite, gene-based association tests

## Abstract

The standalone C++ Mega2 program has been facilitating data-reformatting for linkage and association analysis programs since 2000. Support for more analysis programs has been added over time. Currently, Mega2 converts data from several different genetic data formats (including PLINK, VCF, BCF, and IMPUTE2) into the specific data requirements for over 40 commonly-used linkage and association analysis programs (including Mendel, Merlin, Morgan, SHAPEIT, ROADTRIPS, MaCH/minimac3). Recently, Mega2 has been enhanced to use a SQLite database as an intermediate data representation. Additionally, Mega2 now stores bialleleic genotype data in a highly compressed form, like that of the GenABEL R package and the PLINK binary format. Our new Mega2R package now makes it easy to load Mega2 SQLite databases directly into R as data frames. In addition, Mega2R is memory efficient, keeping its genotype data in a compressed format, portions of which are only expanded when needed. Mega2R has functions that ease the process of applying gene-based tests by looping over genes, efficiently pulling out genotypes for variants within the desired boundaries. We have also created several more functions that illustrate how to use the data frames: these permit one to run the pedgene package to carry out gene-based association tests on family data, to run the SKAT package to carry out gene-based association tests, to output the Mega2R data as a VCF file and related files (for phenotype and family data), and to convert the data frames into GenABEL format. The Mega2R package enhances GenABEL since it supports additional input data formats (such as PLINK, VCF, and IMPUTE2) not currently supported by GenABEL. The Mega2 program and the Mega2R R package are both open source and are freely available, along with extensive documentation, from https://watson.hgen.pitt.edu/register for Mega2 and https://CRAN.R-project.org/package=Mega2R for Mega2R.

## Introduction

During an association or linkage analysis project, one may need to analyze the data with several different programs. Unfortunately, it can often be quite difficult to get one’s data in the proper format required by each different computer program. Not only must the data be converted to the proper format, but also the loci must be reordered into the desired order. Writing custom reformatting scripts can be error-prone and very time-consuming. To address these problems, we created Mega2 ^1, 2^, which can be obtained from the University of Pittsburgh site or via BitBucket. The early Mega2 could read input data in only a few formats: LINKAGE format ^3– 5^ and Mega2 format. The Mega2 format allowed you to specify additional information in a more straightforward way, via a simple tabular format, than can be done with LINKAGE format. The earliest versions of Mega2 were written in the C computer language.

Over time, Mega2 was upgraded to the C++ computer language and some of the internals were rewritten. Reformatting data for more analysis programs was gradually added to Mega2. The formats for genetic data changed over time, and Mega2 was enhanced to read input data in PLINK format ^6^, VCF format ^7^, and IMPUTE2 format ^8– 11^. The volume of genome-wide marker data increased significantly, so Mega2 was extended to support the compression of biallelic genotype data (though still supporting microsatellite and other polymorphic data as non-compressed legacy data). As the volume of genotype data increased, it became slow and tedious to validate the genotype data and generate allele frequency data for each separate analysis that Mega2 was used for. Rather, Mega2 was restructured so that the validation and meta data generation were performed once for a given data set.

Until recently, Mega2 used C language structures to store its intermediate data before analysis. Several alternative strategies were considered to save these data for subsequent reuse without having to recompute, reanalyze, and revalidate the data. The first choice was to dump the raw structure data to disk. Reloading the data is fast but it is much harder to inspect the data for debugging. Also, the internal pointers would have to be relocated to new storage areas. A better solution, for many reasons, was to create a SQLite table for each C structure and “insert” the C structure data into the table. Mega2 later uses this database, via a menu-driven interface, to provide the data needed for any particular analysis. In addition, the data in the database are inherently inspectable and there are database tools to help to selectively extract data. The data are portable and can be shared on different platforms. Since interfaces for many languages are provided for SQLite, the data are accessible in many languages besides C. Of particular interest is R ^12^ because there is a wealth of existing genetic analysis programs already created, well documented, and available in standard repositories such as The Comprehensive R Archive Network (CRAN) and Bioconductor. Also, researchers regularly use R to develop new analysis algorithms because R provides a rich environment for statistical computing with many useful libraries. Indeed, there are are many other R packages that can handle genetic data. For example, VariantAnnotation, seqminer, and SeqArray support efficient processing of VCF/BCF files, while SeqVarTools and GENESIS support association testing and other relevant statistical methods ^13– 17^.

We describe here our Mega2R package, which makes it easy to load SQLite Mega2 databases directly into R as data frames for further analysis and manipulation. In addition, we document several R functions that illustrate how to use the Mega2R data frames as well as perform useful functions: the Mega2pedgene function to run the pedgene R package ^18^ to carry out gene-based association tests on family data using selected marker subsets, the Mega2SKAT function to run the SKAT R package ^19^ to carry out gene-based association tests on family data using selected marker subsets, the Mega2VCF function to output the Mega2R data as a VCF file and related files (for phenotype and family data), and the Mega2GenABEL function to convert the data frames into GenABEL R objects ^20^. Typically, these R functions are designed to process a small collection of markers at a time. Our versions process all the markers in a transcript for as many genes as desired. Alternatively, the transcripts can be abstracted to just a table of chromosome/start/end base pair positions. Mega2R eases this process of looping over genes, efficiently pulling out genotypes for variants within the transcript boundaries. In addition to describing the functionality of our Mega2R package, we also provide a Use Case illustrating how to apply it in practice.

## Methods

### Implementation

**Figure 1.**
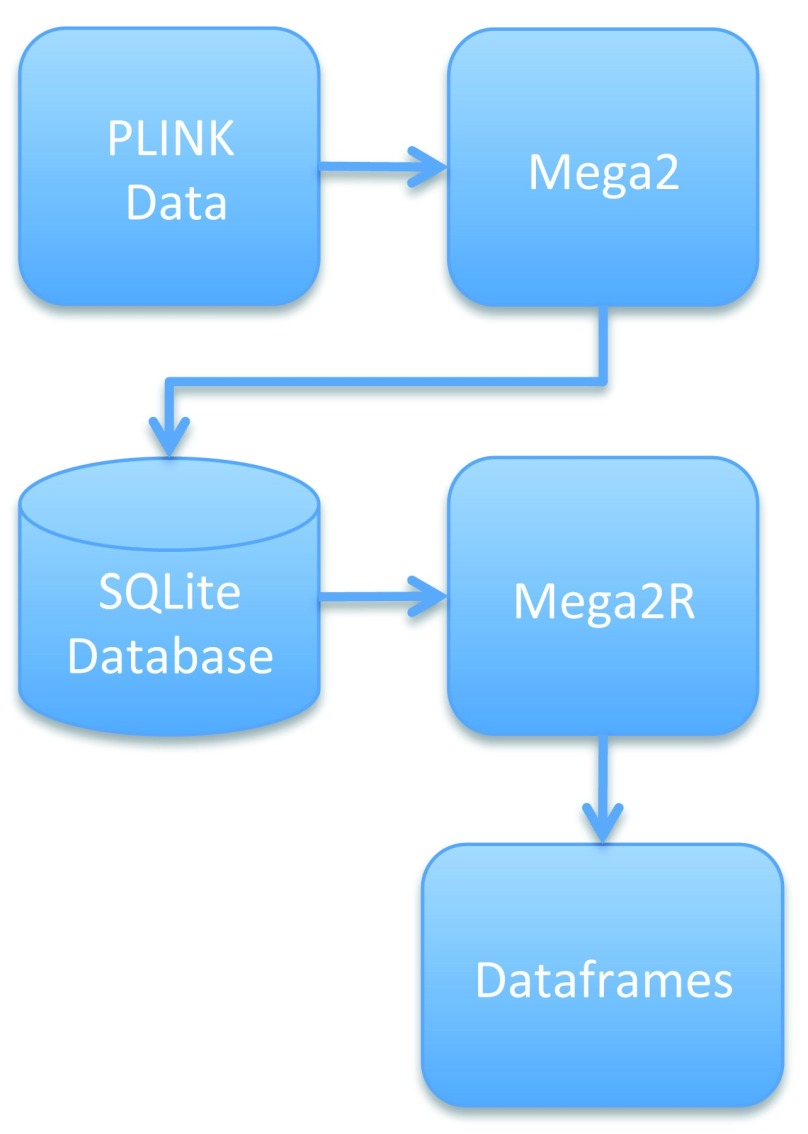
Input data (PLINK in this example) are converted into a SQLite database by Mega2; this database is then read by Mega2R, making the data accessible within R as data frames.

**Figure 2.**
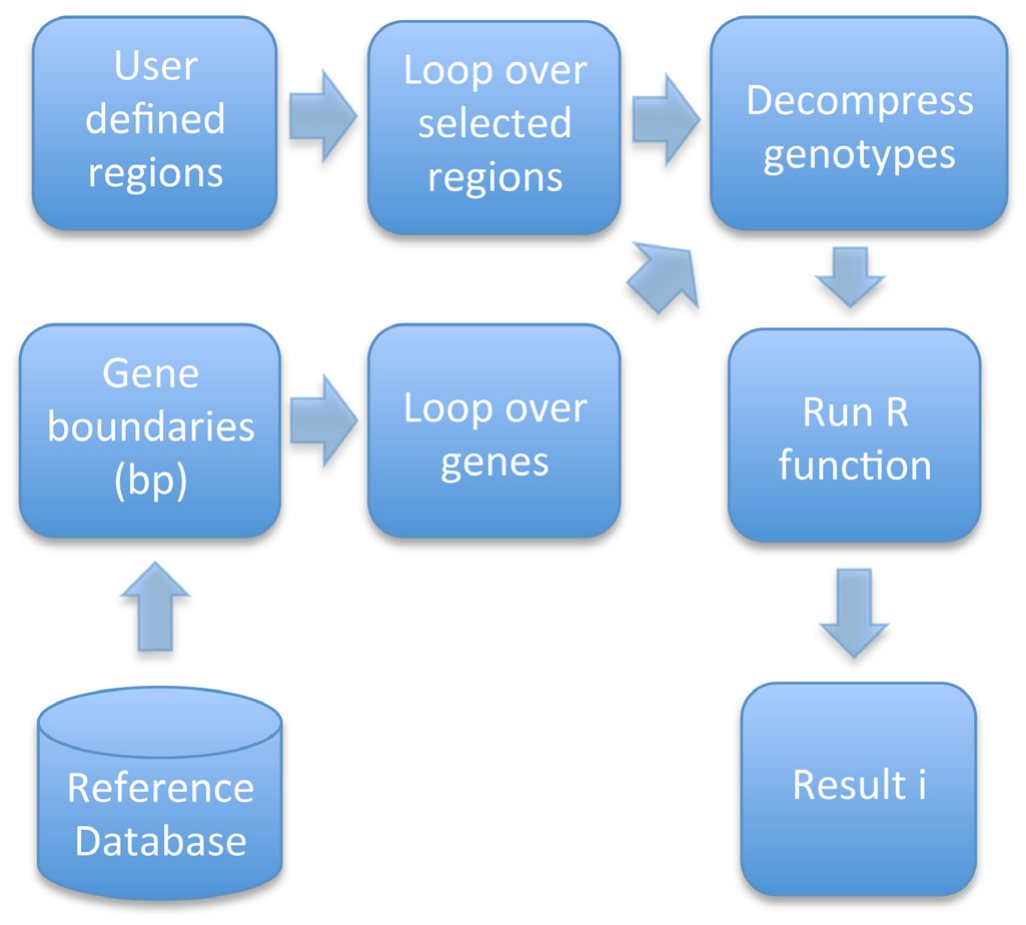
Mega2R provides an efficient and flexible wrapper for iterating through gene regions, running R functions on variants within the desired boundaries.

Mega2R is an R package which loads a Mega2-created SQLite database of genetic information into R as data frames for further analysis ( Figure 1). Parts of Mega2R are written in C++ for speed and efficiency. Mega2R is memory efficient, keeping its genotype data in a compressed format, portions of which are only expanded when needed. Mega2R has functions that ease the process of applying gene-based tests by looping over genes, efficiently pulling out genotypes for variants within the desired boundaries ( Figure 2).

### Operation

The Mega2R package is available from the CRAN repository, and can be run within the R environment on Windows, Mac OS X, and Linux computers. The Mega2R CRAN page documents package dependencies. To prepare an input database for Mega2R, the C++ program Mega2 ^1, 2^ is needed; it can be obtained from the University of Pittsburgh site or via BitBucket.

### Environments

Before we discuss the Mega2R package, we must describe one important convention of how the database information is stored and how it is accessed later. The Mega2R R package function, *read.Mega2DB*, reads the tables needed by Mega2R from a Mega2 SQLite database into a collection of data frames. It returns an R environment containing these data frames. (If you are unfamiliar with environments, you can think of them as data frames. For example, ’ENV’$ **locus_table** will access the **locus_table** variable from the environment, ’ENV’; this is similar to fetching an "observation" from a data frame. The difference is when you change data in an ordinary data frame passed to a function, the change does not affect the original data frame; only the function’s local data is changed and all changes are forgotten when the function exits. If you change the data in a data frame of an environment passed to a function, the change is permanent.)

All Mega2R functions that do not return an environment need to have an environment supplied as an argument; the environment is used to store the data frames that contain the SQLite database. There are two ways to pass the environment to a Mega2R function. If you assigned the result of *read.Mega2DB* to the variable ’seqsimr’, then you could supply the value ’seqsimr’ as the named argument, ’envir’:

           showMega2ENV(envir = seqsimr)

The second choice is a bit of a “hack” but it is very convenient. Every Mega2R function (that does not return an environment) has a named argument, ’envir’, defined to take on the default value ’ENV’, as in

           showMega2ENV = function(envir = ENV) { ... }

The code above, assigns the local variable, ’envir’ the default value ’ENV’. Thus, if ’envir’ is not provided in the function call, R will use the default assignment and look up the value of ’ENV’. This “hack” does not handle the case where ‘ENV‘ is defined in an outer frame, not the global environment. In this situation, we search backwards from the calling frame to find the first ‘ENV‘ and use it.

### The Mega2 SQLite database

In the current Mega2 design, there are SQLite tables for loci data, marker data, allele data, map data, phenotype data, and genotype data as well as pedigree and person data. For completeness, Supplementary File 1 shows all the tables and their fields. Here, we will make a few general observations about the tables. The first issue was mentioned earlier: How should we handle pointers. Where necessary, we added a field to a table, which we call a “link” field, viz. locus_link. This field is a unique integer. When the data are read back in by Mega2 via C++, these links are looked up to find a pointer to use. Consider the **locus_table** and the **allele_table**. They both have a locus_link. The link in the **locus_table** is hashed and a pointer to the corresponding entry in the **locus_table** is stored. When the **allele_table** is read, the locus_link is looked up, then a pointer is inserted into a new field in the “in memory” **locus_table** (using the look up pointer) to point to the memory location of the **allele_table** entry, *i.e*. the **locus_table** in memory will point to the **allele_table**. These “links” are also very useful in R for performing merges of related tables. For example, in the above case, a merge can produce a table with rows containing both locus and allele data. Similar links are used to associate family data with person data, as well as to associate person data with phenotype data and genotype data.

Most tables are composed of integer, numeric and string data (see Supplementary File 1). The phenotype table is an exception. The normal trick of adding an extra column ( *e.g.*, the phenotype id) per row to indicate which phenotype’s data is contained in the row becomes more complicated because affection status traits and quantitative traits have different data types and number of values. Instead, the phenotype data for each person is stored as one long raw vector composed of multiple values with 8 bytes of data per value either representing an affection status value or a quantitative value. Similarly, the genotype data are stored as a raw vector with a pair of bytes representing a genotype for non bialleleic markers and a raw compressed bit vector with two bits per genotype for bialleleic markers. Note that the internal compressed genotype vector used by Mega2 is one long vector, while the genotype data written to the database is split into separate vectors for each chromosome. The end of each vector may have 0, 1, 2, or 3 unused bit pairs representing no markers. When reading back in from the database, the Mega2 C++ program reassembles the vectors without these gaps. The R version is not as sophisticated: it reassembles all the chromosome vectors and includes these gaps. It then takes care when referencing the n ^th^ marker, to add in the gaps of all the chromosomes that come before the n ^th^ marker’s chromosome. Note that reassembling the data with no “virtual markers” would require a lot of bit manipulations in R which would not be terribly efficient; while keeping track of the virtual markers introduced by the gaps is not particularly hard.

### The Mega2R package base functions

After using Mega2 to compress and store one’s genetic data in a Mega2 SQLite database, then one can use the Mega2R *read.Mega2DB* function to read the database into R data frames within the specified R environment.

library(Mega2R)
ENV = read.Mega2DB("seqsimr.db", verbose = TRUE)

If the verbose flag is true, summary statistics will be printed as the data frames are created. After running the *read.Mega2DB* function, the ENV environment will contain a set of data frames ending in “ **_table**”, viz. **person_table**, **locus_table**. These contain raw data copied directly from the Mega2 SQLite database (although some of the original fields that are only needed for Mega2 are not copied).

The *read.Mega2DB* function also creates other data frames containing derived information. The function *mkfam* is called by *read.Mega2DB* to create the **fam** data frame, the equivalent of a PLINK .fam sample information file. It merges the rows from the **pedigree_table**, **person_table**, and **phenotype_table** to make a **fam** data frame. The **pedigree_table** has a pedigree_link, **person_table** has a pedigree_link and person_link, and **phenotype_table** has a person_link field. These link fields are used by the R ’merge’ function to assemble the data. Later, you may want to prune the **fam** data frame to restrict the samples processed. For, example you might want to eliminate samples with unknown case/control status. After you prune the **fam** information, the function, *setfam*, will set the **fam** data frame while also appropriately pruning the **phenotype_table** and **unified_genotype_table** to contain only those persons left in the **fam** data frame.

The **unified_genotype_table** collects all the raw byte vectors from the genotype_table stored in the input SQLite database by chromosome for each person and concatenates them into a new vector for each person. The concatenation can introduce up to three “virtual markers” (as mentioned above) at the end of each chromosome if the number of markers contained is not an exact multiple of 4 (genotypes per byte). The **marker_table** contains the offset of the marker genotype data assuming all the markers across all the chromosomes are contiguous. There is an analogous offset column needed for the **unified_genotype_table** accounting for the “virtual markers” in the gaps; this offset column is computed and added to the **marker_table**. The **markers** data frame is a subset of the **marker_table** data containing only the name, chromosome, position, original **marker_table** offset and marker offset into the **unified_genotype_table** vector.

### Mega2R marker range functions

We might have genotype data for thousands of samples and millions of markers in a study, but we are unlikely to have the memory to support the associated genotype matrix in R. The Mega2 genotype data is compressed in the SQLite database and remains compressed in its data frame. Most of the time, we do not need to decompress all of it at once. We typically want to examine the markers on a specific chromosome or in the region of some genes of interest. We only decompress the set of markers we need. The key idea is we want to find the set of markers that lie within some base pair range and process just those markers and then we repeat this for the “next” range. We provide two ways to specify these ranges, via the *setRanges* and *setAnnotations* functions. In addition, we want to iterate through multiple ranges as would appear in a set of selected gene transcripts and process the markers. To do this, we process each range of markers by invoking a callback function on the range, markers and related data, via one of the *applyFnToRanges*, *applyFnToGenes*, or *applyFnToMarkers* functions.

### The *setRanges* and *applyFnToRanges* functions

One of Mega2R’s strengths is its ability, via the *applyFnToRanges* and *applyFnToGenes* functions, to loop over a list of ranges or genes, for each one selecting markers that lie within the region/gene of interest. To do this, one has to have well-defined gene boundaries. By default, Mega2R provides an internal list of the chromosome and base pair ranges for the transcripts defined in the refGene table from the UCSC Genome Browser reference assembly GRCH37. The list was modified to eliminate multiple records of the same gene with the exact same transcript start and transcript end. The list contains 29,062 records. But there are several reasons that the default ranges may not be to your liking. The *setRanges* function lets you use custom ranges; it has two arguments: a range data frame that specifies at least the chromosome, the start position, end position (and optionally a name) of each range, and an index vector of three integers that indicates which columns in the range data frame contain the chromosome, start position and end position. If the range data frame also contains a name column, the index vector should have a final fourth integer that indicates the column containing the name.

The *applyFnToRanges* function (and also the *applyFnToGenes* function, described below) take an initial argument that is a callback function that is called repeatedly for each transcript that has markers present. They also take a final argument that is an R environment. The *applyFnToRanges* function, with no additional arguments, will iterate through each row of the default Mega2R ranges, determine the markers contained between the start and end and then invoke a callback function. Alternatively, *applyFnToRanges* may be given a range argument and a selector argument which will cause it to use the ranges supplied rather than the default. These two arguments are the same format as the corresponding arguments provided to *setRanges*.

The *applyFnToRanges* function requires a callback function of three arguments: the first is a data frame of the markers in the range, the second is a single row from the data frame that defines the range (minimally it has a name, ID, start position, end position, and chromosome), and the third argument is the R environment that contains all the Mega2R data frames. The callback function can apply whatever analysis is required and it can access the other data frames available in the R environment and even store intermediate results back into the R environment. Typically, the callback function will call either *getgenotypes* or *getgenotypesraw* to get a matrix of genotypes values for the markers in the range.

### The *setAnnotations* and *applyFnToGenes* functions

Mega2R can also load a list of genes and their transcripts from the Bioconductor data annotations: “org.Hs.eg.db”and “TxDb.Hsapiens.UCSC.hg19.knownGene”. The former translates a gene name or alias to the entrez ID of the gene. The latter gives start position, end position, and chromosome for each transcript known for every gene. You might want to use the transcript data from another build that is available in Bioconductor or follow their instructions to make your own mapping. The Mega2R function, *setAnnotations*, lets you use alternate tables. Mega2R will load the selected tables when you access the *applyFnToGenes* function.

The *applyFnToGenes* function specifies a list of genes (via the genes_arg argument) from which to extract the transcripts and the corresponding ranges (If the special gene name, “*”, is passed as the only gene argument, the ranges for all transcripts will be chosen). Alternatively, *applyFnToGenes* supports other ways to indicate transcript ranges: on sub-ranges of chromosomes (via the ranges_arg argument), on the entire chromosome (via the chrs_arg argument), or on an arbitrary list of markers (via the markers_args argument). The callback function of three arguments (markers, range, environment) and a list of ranges is passed to *applyFnToRanges* and processed as described above.

### The *applyFnToMarkers* function

The *applyFnToMarkers* function is the simplest function that uses the aforementioned callback function. It takes one argument that contains selected rows of the **markers** data frame. It then invokes the callback function with the marker argument data frame, a NULL range, and the R environment (The range is NULL because there is no range information; this implies that the callback function for *applyFnToMarkers* must check for a range value of NULL).

### The *getgenotypes* and *getgenotypesraw* functions

The functions *getgenotypes* and *getgenotypesraw* return a matrix of nucleotide pairs or a matrix of encoded integers with a column for each marker and containing a row per sample. Both functions take two arguments: rows of the **markers** data frame and an environment. The first argument is usually computed by one of the *applyFnToRanges*, *applyFnToGenes*, or *applyFnToMarkers* functions. The **markers** data frame has two offsets that are used internally by the decompression code; it also has a name, chromosome number and position that can be used to identify a marker to the user. The two *getgenotypes* functions are dispatches that call Rcpp code. The R code collects genotype and allele data from the R environment’s data frames and passes these data arguments to the Rcpp code; it also calls the proper function for the level of compression: 2 bits or 2 bytes per marker. For each marker specified, *getgenotypes* returns for each sample a string of the corresponding pair of nucleotides, while *getgenotypesraw* returns for each sample a single integer with the integer for the first allele shifted 16 bits and or’ed to the integer for the second allele.

### The *Mega2pedgene* function

We now give a brief overview of several processing functions that Mega2R makes available and that illustrate how to build new functions using Mega2R. A much more detailed example may be found in the “Use Case” section.

The ’pedgene’ R package performs gene-level association tests with disease status for pedigree data ^18^. Mega2R enhances ’pedgene’ via the *Mega2pedgene* function, which automates and eases computation of the ’pedgene’ statistics for all the genes in the genome. The *Mega2pedgene* function does this by invoking *applyFnToRanges* on the specified ranges or calls *applyFnToGenes* on gene names. Before running the *Mega2pedgene* function, we first call *init_pedgene* rather than *read.Mega2DB*; the former uses the function *dbmega2_import* to load the Mega2 database. Then it generates a modified **fam** data frame, a kind of family file where the case/control values of 0/1/2 are translated to NA/0/1, as required by *pedgene*. The *pedgene* package requires recoding of the genotypes 1/1, 1/2 (and 2/1), and 2/2 (with raw encodings: 65537, 65538 (and 131073), 131074) to 0, 1, and 2 respectively; plus the alleles must be flipped as needed so that 1 is the major allele. The callback function, *DOpedgene*, transforms the raw genotype matrix and then it calls the *pedgene* function with several different marker weights ( *e.g.*, unweighted, Madsen-Browning weights, and *β* density weights). It returns the p-value of the kernel and burden association statistics for each weight. The results are appended to a data frame in the environment, pedgene_results.

### The *Mega2SKAT* function

If one has a sample of unrelated individuals, the *Mega2SKAT* function eases the process of applying the region-based association tests that are implemented in the SKAT package ^19, 21, 22^. The SKAT package uses kernel regression approaches to compute association statistics such as the Sequence Kernel Association Test (SKAT) and its optimized version (SKAT-O), both for quantitative and dichotomous traits. The *Mega2SKAT* function is rather similar to *Mega2pedgene* (above). The *init_SKAT* function behaves very much like *init_pedgene;* in addition, it computes a phenotype data frame. The *Mega2SKAT* function has the signature:

             Mega2SKAT = function (f, ty, gs = 1:100, genes = NULL, skat = SKAT::SKAT,
            envir = ENV, ...)

The parameters **f** and **ty** specify the formula and phenotype type that are used to invoke *SKAT_Null_model*. The **skat** argument indicates the particular SKAT package function to call and the **...** arguments are place holders for any additional arguments that the chosen **skat** function needs.

### The *Mega2VCF* function

The VCF data format was originally defined by the 1000 Genomes Project ^23^ for data storage ^24^. The current version of data format is available from GitHub ^25^. The *Mega2VCF* function serves several purposes. It allows VCF format files to be generated for analysis by programs that require VCF input. It shows how to extract data provided in the Mega2R data frames for subsequent analysis by other R packages. Finally, it provides a regression for Mega2R by producing the same files from an SQLite database via R code that Mega2 can produce from the identical database via C++ code. The VCF file and related files created both ways should be the same except for time stamps.

Before running the *Mega2VCF* function, you should first call the *read.Mega2DB* function to load a Mega2 database. A .vcf VCF file is the main output of *Mega2VCF*; it is created from the genotype matrix, supplemented by columns from the **fam** table and **allele_table** table. Besides the .vcf file, *Mega2VCF* generates several additional complementary files. Below, we indicate the file suffix, the internal function that generates the file, and the file contents are presented in Table 1.

**Table 1.** *Mega2VCF* output file suffix, function to generate the file, and contents of the file.

file suffix	function	contents
**.vcf**	*Mega2VCF* *mkVCFhdr*	contains the multi column VCF file with header; each row contains genotypes for all the samples for that marker
**.fam**	*mkVCFfam*	contains the 6 columns of the .fam file
**.freq**	*mkVCFfreq*	contains the allele frequency information
**.map**	*mkVCFmap*	contains the supplied maps with their genetic or physical distances
**.phe**	*mkphenotype*	contains the phenotype information: quantitative and affection status
**.pen**	*mkVCFpen*	contains the penetrance information

The code in each function illustrates how to assemble the corresponding data from the Mega2R data frames. The *mkphenotype* function is a bit subtle. The database contains a raw vector of 8 bytes times the number of phenotypes (per person). The 8 bytes encode either one double precision float number or two integers depending on the encoding of the trait phenotype in the **locus_table** (quantitative or affection status). The *readBin* function is used to convert the raw bytes in each 8 byte vector to the correct form.

Another interesting aspect of the code design is how we compute genotype data for a large numbers of markers without needing large amounts of memory. We build the .vcf file a chunk at a time, where a chunk contains a relatively small number of markers (currently 1,000). We generate the genotype matrix for a chunk then append the results for the chunk (of markers) to the .vcf file. Then we get a new chunk and repeat. This looping strategy might prove useful for other situations where the results can be written incrementally, a block of markers at a time.

### The *Mega2GenABEL* function

The GenABEL ^20^ package is available in the archive section of CRAN ( https://CRAN.R-project.org/package=GenABEL). The GenABEL package provides many functions for genome-wide association analysis and it accepts data in several formats. But Mega2 accepts input in still more formats, notably VCF, PLINK, IMPUTE2 and even Linkage format. Thus *Mega2GenABEL* can be a bridge to easily convert data for GenABEL analysis.

Before running the *Mega2GenABEL* function, you must call the *read.Mega2DB* function to load your database and you should save the returned environment into ’ENV.’ The function, *Mega2GenABEL*, returns a gwaa.data-class object for the selected markers. It operates by re-writing the Mega2R data frames into one of the input formats that GenABEL supports; currently, this is PLINK .tped format. This requires a .tped file, a .fam file, and a .phe file; they are stored in a temporary directory. The .tped file looks very much like a VCF .vcf file with entries in each marker’s row for the samples’ genotype data. (But, in comparison to the .vcf file, there are fewer fields of metadata in a .tped file and the genotype data specify real allele labels rather than VCF’s REF/ALT field references.) *Mega2GenABEL* calls the GenABEL “glue” functions, *convert.snp.tped*, to process the a .tped file and .fam file into a generic GenABEL “raw” format file, then it converts that raw format file and a phenotype file into a GenABEL gwaa.data-class object via the function, *load.gwaa.data*, and then it deletes the temporary files.

The function *Mega2ENVGenABEL* produces the same object as *Mega2GenABEL*. It does not write out any temporary files, but rather converts the Mega2R genotype and related data in memory to a GenABEL gwaa.data-class object. It is mainly written in Rcpp and runs 10 to 20 times faster than the *Mega2GenABEL* function.

## Use case

We now show some examples of how to use Mega2R. First, we illustrate the base functionality of the Mega2R package ( *e.g.*, iterating over gene ranges and getting genotypes from the database). Then we demonstrate one of the extended functions, *Mega2pedgene*. The other extended functions, *Mega2SKAT*, *Mega2VCF* and *Mega2GenABEL* are illustrated in the Mega2R package vignette. Further, details of all the Mega2R functions are available in the Mega2R manual Finally, the package source can be obtained from CRAN or it can be obtained via git from Bitbucket.

The R code in the examples below was executed during the creation of this document using the knitr R package (version 1.20) ^26– 28^.

### Simulated data

We used the SeqSIMLA2 ^29^ program, which is available from SourceForge, to simulate an example dataset. To do this, we started with the first example under the “Prevalence” tab within the “Simulate by Disease status” tab on the tutorial page. This simulation creates 1,380 samples of 500,000 markers on a subrange of chromosome 1. To illustrate the Mega2 and Mega2R operations that follow, we subsampled the data down to 1,380 people and 1,000 polymorphic markers.

### Installation

***R.*** We will assume that you have started an R session in which you type the commands in this Use Case (We used R version 3.5.0).

You should first install the package Mega2R.

install.packages("Mega2R")

***Bioconductor.*** Below we will use ’pedgene’ carry out gene-based association tests, where ’genes’ are defined according to a database containing the boundaries of the gene transcripts. This requires two Bioconductor Annotations databases to be installed. The first line below loads the Bioconductor biocLite loader and the next two lines install two annotation databases. One annotation database provides the gene transcript locations and the other maps gene names to Entrez gene IDs. (As described in the section about pedgene below, you may choose a different transcript database from Bioconductor or construct one of your own.) Please type in R:

source("https://bioconductor.org/biocLite.R")
biocLite("TxDb.Hsapiens.UCSC.hg19.knownGene")
biocLite("org.Hs.eg.db")

The above steps are run once.

***Use case data.*** First, you will need to load the Mega2R package. Type:

library(Mega2R)

The files you will need for this Use Case are provided in the Mega2R package. You need to extract these files to the current directory via this command:

dump_mega2rtutorial_data(".")

You should see the following files:

list.files(where_mega2rtutorial_data("."))

##  [1] "MEGA2.BATCH.seqsimr" "MEGA2.BATCH.srdta"   "MEGA2.BATCH.vcf"
##  [4] "Mega2r.map"          "Mega2r.map.gz"       "Mega2r.ped"
##  [7] "Mega2r.ped.gz"       "seqsimr.db"          "seqsimr.db.gz"
## [10] "srdta.db"            "srdta.db.gz"

Note: The use of the “mega2” executable in our examples expects the Mega2.BATCH.<name> files to be in the working directory and the latter files expect their data files to be in the working directory. When you are done with these exercises, the “clean” command will remove these files and other temporary files:

clean_mega2rtutorial_data(".")

### Using Mega2R to access and process genetic data

***Creating a Mega2 database.*** We have provided files in this package that contain the data from the simulation. These files are in PLINK ped format data:

• Mega2r.ped
• Mega2r.map

If you do not wish to install Mega2 right now, you can skip this database creation step and instead later use the seqsimr.db database that was placed in your directory by the *dump_mega2rtutorial_data(“.”)* command. You can obtain the Mega2 program from the University of Pittsburgh site. Then, you will invoke Mega2 on your data. To make matters simple, we will use a pre-constructed Mega2 batch file to automate the processing by Mega2. To run Mega2 to process and create the ’SQLite’ database ’seqsimr.db’, we issue this command at the Unix prompt from within the directory containing the Use Case example files:

          mega2 MEGA2.BATCH.seqsimr

Note: The vignette associated with the Mega2R package illustrates what the results of this Mega2 run would look like.

If you do not provide the command-line argument giving the name of a BATCH file, Mega2 will proceed to ask a series of interactive questions to collect the information needed to produce a database. In addition, it will create a Mega2.BATCH file, similar to the one we suggested you use. You can look at the “Quick Start” section of the Mega2 documentation to better understand the interactive process.

The MEGA2.BATCH.seqsimr file begins with a rather long comment section indicating the keyword values that may be set and their default values. Towards the end of the file, we set the inputs to **Mega2r.ped** and **Mega2r.map**, indicate the input is PLINK ped format with parameters, and indicate that Mega2 should produce a database called **seqsimr.db**, etc. (The initial comment section is not shown and unimportant lines are elided.)

Input_Database_Mode=1
Input_Format_Type=4
Input_Pedigree_File=Mega2r.ped
Input_PLINK_Map_File=Mega2r.map
...
PLINK_Args= --cM --missing-phenotype -9 --trait default
...
Value_Marker_Compression=1
Analysis_Option=Dump
...
DBfile_name=seqsimr.db
...

If you wish to use any of the Mega2R functions described here on your own data, you will have to first run “mega2” to convert your data into an ’SQLite’ database.

### Reading and examining a Mega2 database

The Mega2R package facilitates reading genetic data from a Mega2-created SQLite database.

**Reading a Mega2 database** After you have created the SQLite database “seqsimr.db”, start up R, load the Mega2R package, then use the function *read.Mega2DB* to read a Mega2 database.

library(Mega2R)
ENV = read.Mega2DB("seqsimr.db", verbose = FALSE)

The first argument should be the path to the database. If ’verbose’ is TRUE, for each data frame created in ’ENV’, *read.Mega2DB* emits two lines: one with the number of rows and number of columns of the data frame and the other with the column names of the data frame. Finally, an ’environment’, which contains the data frames, is returned.

**Verbose Flag** When verbose is set in the initial read.Mega2DB, the value will be remembered. It may be used by any subsequent function. If verbose is TRUE, Mega2R functions will print diagnostic information.

**Use of an Environment** The environment returned is used to store the data frames that contain the ’SQLite’ database. The code above stores the environment in global variable ’ENV’. If the named argument, ’envir’, is not provided in any subsequent Mega2R function call, R will look up the value of ’ENV’ starting at the calling frame and chaining up the call stack to the global environment.

**Back to more examples.** The ’ls(ENV)’ function will show you all the variables in the ’ENV’ environment. (You probably have used it without arguments to show you the variables in the global environment .GlobalEnv.) Type:

ls(ENV)

##  [1] "affectclass_table"      "allele_table"
##  [3] "charstar_table"         "chr2int"
##  [5] "DBcompress"             "DBMega2Version"
##  [7] "dosage"                 "dosageRaw"
##  [9] "int_table"              "locus_allele_table"
## [11] "locus_table"            "LocusCnt"
## [13] "map_table"              "mapnames_table"
## [15] "MARKER_SCHEME"          "marker_table"
## [17] "markers"                "Mega2R"
## [19] "pedigree_brkloop_table" "pedigree_table"
## [21] "person_brkloop_table"   "person_table"
## [23] "PhenoCnt"               "phenotype_table"
## [25] "positionVsName"         "refIndices"
## [27] "refRanges"              "traitaff_table"
## [29] "txdb"                   "unified_genotype_table"
## [31] "verbose"

A more informative overview of the database can be had with:

showMega2ENV()

## locus count:   1001; phenotype count:  1; compression: 2 bits
## marker count:  1000; sample count:  1380
##
## genetic and physical maps:
##   map name map number
## 1      Map          0
## 2       BP          1
##
## Phenotypes:
##   Index    Name      Type
## 1     1 default affection
##
##
## basic tables:
##                        rows cols
## affectclass_table         1    9
## allele_table           2002    5
## charstar_table            5    3
## int_table                35    3
## locus_table            1001    5
## map_table              2000    6
## mapnames_table            2    6
## marker_table           1000    8
## pedigree_brkloop_table   20    8
## pedigree_table           20    8
## person_brkloop_table   1380   11
## person_table           1380   11
## phenotype_table        1380    4
## traitaff_table            1    4
##
## derived tables:
##                        rows cols
## fam                    1380    8
## markers                1000    5
## unified_genotype_table 1380    2

**Use standard R operations to examine the created data frames** Try typing:

str(ENV$locus_table)

## 'data.frame': 1001 obs. of  5 variables:
##  $ pId       : int  1 2 3 4 5 6 7 8 9 10 ...
##  $ LocusName : chr  "default" "snp1" "snp2" "snp3" ...
##  $ Type      : int  2 4 4 4 4 4 4 4 4 4 ...
##  $ AlleleCnt : int  2 2 2 2 2 2 2 2 2 2 ...
##  $ locus_link: int  0 1 2 3 4 5 6 7 8 9 ...

### Iterating through Gene Transcripts

Mega2R provides two ways to compute a function on the genotypes (or markers) in each of the transcripts. These are further illustrated via examples in section about ’pedgene’ below.

**setRanges**    The default ranges contains 29,062 records taken from the UCSC Genome Browser reference assembly GRCH37. We show a bit of the data frame below. Each row contains 5 values: a transcript id, the gene id and three position values: chromosome, start base pair and end base pair.

 dim(ENV$refRanges)

## [1] 29062     5

 head(ENV$refRanges, 3)

##          XX    SYMBOL TXCHROM   TXSTART     TXEND
## 1 NM_005286    NPBWR2   chr20  62737182  62738184
## 2 NR_026775 LINC00240    chr6  26924771  26991753
## 3 NM_007188     ABCB8    chr7 150725509 150744869

You may load your own range set instead of the default. You create a data frame that lists, for each range, the chromosome, the start position, and the end position. And you create an integer index vector that indicates which column contains which data. These two become the arguments to the *setRanges* function as shown in the example below. When the index vector contains only three entries, a range name is generated by concatenating the position information and adding it to the range data frame.

 ranges = matrix(c(1, 2240000, 2245000,
                      1, 2245000, 2250000,
                      1, 3760000, 3761000),
                      ncol = 3, nrow = 3, byrow = TRUE)

 setRanges(ranges, 1:3)

 dim(ENV$refRanges)

## [1] 3 4

 head(ENV$refRanges)

##   X1      X2      X3          ChrStartEnd
## 1  1 2240000 2245000 chr1:2240000-2245000
## 2  1 2245000 2250000 chr1:2245000-2250000
## 3  1 3760000 3761000 chr1:3760000-3761000

If you provide an index vector with four entries, the fourth one indicates the column containing the names of the ranges.

 ranges = matrix(c(1, 2240000, 2245000,
                      1, 2245000, 2250000,
                      1, 3760000, 3761000,
                      1, 3761000, 3762000),
                      ncol = 3, nrow = 4, byrow = TRUE)
 ranges = data.frame(ranges)
 ranges$name = LETTERS[1:4]
 names(ranges) = c("chr", "start", "end", "name")

 setRanges(ranges, 1:4)
 dim(ENV$refRanges)

## [1] 4 4

 head(ENV$refRanges)

##  chr    start      end name
## 1  1  2240000  2245000    A
## 2  1  2245000  2250000    B
## 3  1  3760000  3761000    C
## 4  1  3761000  3762000    D

**applyFnToRanges**    The function, *applyFnToRanges*, goes through each range and finds the markers that fall within the bounds. The first argument of the *applyFnToRanges* function is a callback function with three arguments: the markers in range, the selected range entry and the environment. The callback is invoked repeatedly for each transcript range that contains any markers. If there are no markers contained and the ’verbose’ flag is set, a warning will be printed. The callback function builds a genotype matrix for the samples and each marker in the range. If necessary, the environment (’envir’) can be used to store information between successive invocations.

For the examples that follow, we use “show” as the callback function, which simply prints, for each range, the range itself, the first three lines of the markers within the range, and the first three lines of the genotype matrix, in that order. It also prints a little banner before each argument. Finally, it does not print the environment argument value; it does not change.

show =  function(m, r, e) {
     print("** Ranges **")
     print(r)
     print("** Markers **")
     print(head(m,3))
     print("** Genotypes (individuals in rows, markers in columns) **")
     print(head(getgenotypes(m),3))
}

A simple example is shown below using the ranges value that were most recently set. We see that the range named “A” contains markers in our example data set.

     ENV$verbose = FALSE
     applyFnToRanges(show)

## [1] "** Ranges **"
##   chr   start     end   name
## 1   1 2240000 2245000      A
## [1] "** Markers **"
##    locus_link  locus_link_fill   MarkerName  chromosome    position
## 57         57               57     snp22730           1     2243896
## 58         58               58     snp22731           1     2243897
## 59         59               59     snp22733           1     2243899
## [1] "** Genotypes (individuals in rows, markers in columns) **"
##      [,1] [,2] [,3] [,4] [,5] [,6] [,7] [,8]
## [1,] "12" "12" "11" "12" "12" "22" "11" "11"
## [2,] "12" "11" "11" "12" "12" "12" "11" "11"
## [3,] "11" "12" "11" "11" "11" "11" "11" "11"

*applyFnToRanges* can also be provided explicit ranges by using the ’ranges_arg’ argument:

applyFnToRanges(show,
                  ranges_arg =
                  matrix(c(1, 4000000, 5000000, "range4m",
                            1, 5000000, 6000000, "range5m",
                            1, 6000000, 7000000, "range6m",
                            1, 7000000, 8000000, "range7m",
                            1, 8000000, 9000000, "range8m",
                            1, 9000000,10000000, "range9m"),
                           ncol = 4, nrow = 6, byrow = TRUE),
                  indices_arg = 1:4)

**setAnnotations**    If you are iterating/selecting via genes, the default transcript database is “TxDb.Hsapiens.UCSC.hg19.knownGene” from Bioconductor; it is stored in the environment as shown below:

 ENV$txdb
 ENV$entrezGene

## [1] "TxDb.Hsapiens.UCSC.hg19.knownGene"
## [1] "org.Hs.eg.db"

Of course, you can change this database. Suppose we want to use build “hg18”, we would run:

 setAnnotations("TxDb.Hsapiens.UCSC.hg18.knownGene", "org.Hs.eg.db")

 ENV$txdb
 ENV$entrezGene

## [1] "TxDb.Hsapiens.UCSC.hg18.knownGene"
## [1] "org.Hs.eg.db"

**applyFnToGenes**    The function *applyFnToGenes* can apply the ’show’ function to the transcripts of a particular gene via the genes_arg argument. For example, here we apply the ’show’ function to transcripts of the *CEP104* gene:

    # Switch back to using build hg19
    setAnnotations("TxDb.Hsapiens.UCSC.hg19.knownGene", "org.Hs.eg.db")
    applyFnToGenes(show, genes_arg = c("CEP104"))

##
## ’select()’ returned 1:1 mapping between keys and columns
## ’select()’ returned 1:many mapping between keys and columns

## [1] "** Ranges **"
##   ENTREZID  ALIAS SYMBOL TXID      TXNAME  TXCHROM TXSTRAND  TXSTART   TXEND
## 1     9731 CEP104 CEP104 4281  uc001aky.2        1        -  3728645 3773797
## [1] "** Markers **"
##    locus_link locus_link_fill  MarkerName    chromosome position
## 65         65              65    snp24037             1  3762181
## 66         66              66    snp24039             1  3762183
## 67         67              67    snp24041             1  3762185
## [1] "** Genotypes (individuals in rows, markers in columns) **"
##      [,1] [,2] [,3] [,4] [,5] [,6] [,7] [,8]
## [1,] "12" "12" "11" "11" "12" "11" "11" "12"
## [2,] "22" "11" "12" "22" "11" "22" "22" "11"
## [3,] "11" "11" "11" "11" "11" "11" "11" "11"

The *applyFnToGenes* function has several other optional arguments that can request complete chromosomes, (multiple) ranges of base pairs on chromosomes, or collections of markers. All these arguments define ranges that are passed to *applyFnToRanges* for evaluation. Note: If the *genes_arg* argument is set to the special "gene" string "*", then all transcripts in the Bioconductor database, will be processed.

     applyFnToGenes(show, ranges_arg =
                                matrix(c(1,  5000000, 10000000,
                                          1, 10000000, 15000000),
                                         ncol = 3, nrow = 2, byrow = TRUE))

## [1] "** Ranges **"
##   ENTREZID ALIAS           SYMBOL TXID TXNAME TXCHROM TXSTRAND TXSTART
## 1        -     - chr1:5e+06-1e+07    0      -       1        -   5e+06
##   TXEND
## 1 1e+07
## [1] "** Markers **"
##    locus_link locus_link_fill MarkerName chromosome position
## 73         73              73   snp30480          1  8264348
## 74         74              74   snp30484          1  8264352
## 75         75              75   snp30487          1  8264355
## [1] "** Genotypes (individuals in rows, markers in columns) **"
##      [,1] [,2] [,3] [,4] [,5] [,6] [,7] [,8]
## [1,] "11" "11" "11" "11" "11" "11" "11" "11"
## [2,] "11" "11" "11" "11" "11" "11" "11" "11"
## [3,] "11" "11" "11" "11" "11" "11" "11" "11"
## [1] "** Ranges **"
##   ENTREZID ALIAS             SYMBOL TXID TXNAME TXCHROM TXSTRAND TXSTART
## 2        -     - chr1:1e+07-1.5e+07    0      -       1        -   1e+07
##     TXEND
## 2 1.5e+07
## [1] "** Markers **"
##    locus_link locus_link_fill MarkerName chromosome position
## 81         81              81   snp34070          1 12812974
## 82         82              82   snp34071          1 12812975
## 83         83              83   snp34074          1 12812978
## [1] "** Genotypes (individuals in rows, markers in columns) **"
##      [,1] [,2] [,3] [,4] [,5] [,6] [,7] [,8]
## [1,] "11" "11" "11" "12" "22" "22" "22" "11"
## [2,] "11" "11" "11" "11" "12" "12" "12" "11"
## [3,] "11" "11" "11" "11" "22" "22" "22" "11"

    # apply function "show" to all genotypes at the 'snp1' marker
    # NOTE: Since we are using an arbitrary collection of markers, the
    # range is not available.
    applyFnToGenes(show, markers_arg = "snp1")
    
## [1] "** Ranges **"
## NULL
## [1] "** Markers **"
##   locus_link locus_link_fill MarkerName chromosome position
## 1          1               1       snp1          1        2
## [1] "** Genotypes (individuals in rows, markers in columns) **"
##      [,1]
## [1,] "11"
## [2,] "11"
## [3,] "12"

    # apply function "show" to all genotypes on chromosomes 24 and 26
    # remember our example database is only chr 1
    applyFnToGenes(show, chrs_arg=c(24, 26))

### Getting the genotypes

**Encoded Genotypes**    The heart of the above two functions is the calculation of the genotype matrix of the samples. We will build the matrix for the first 10 markers. Remember our database has 1,380 samples so we will just show the *head* of the matrix.

 genotype = getgenotypes(ENV$markers[1:10,])
 dim(genotype)

## [1] 1380   10

 head(genotype, 3)

##      [,1] [,2] [,3] [,4] [,5] [,6] [,7] [,8] [,9] [,10]
## [1,] "11" "11" "11" "11" "11" "11" "11" "11" "12" "12"
## [2,] "11" "11" "11" "11" "11" "11" "11" "11" "22" "11"
## [3,] "12" "11" "11" "11" "11" "11" "11" "11" "22" "11"

**Raw Genotypes**    The function *getgenotypesraw* will be called with the same 10 markers as above. Recall it returns an integer encoding for each genotype. The integer’s high 16 bits are the index for allele 1 and the low 16 bits are the index for allele 2.

# two ints in upper/lower half integer representing allele
 raw = getgenotypesraw(ENV$markers[1:10,])
 dim(raw)

## [1] 1380   10

 head(raw, 3)

##       [,1]  [,2]  [,3]  [,4]  [,5]  [,6]  [,7]  [,8]   [,9]  [,10]
## [1,] 65537 65537 65537 65537 65537 65537 65537 65537  65538 65538
## [2,] 65537 65537 65537 65537 65537 65537 65537 65537 131074 65537
## [3,] 65538 65537 65537 65537 65537 65537 65537 65537 131074 65537

raw.allele.representation = c("0x10001", "0x10002", "0x20001", "0x20002")
print(as.numeric(raw.allele.representation))

## [1]  65537  65538 131073 131074

### How to use applyFnToGenes to apply a user-defined statistical test

The example “show” function used above does not compute any statistics; it just shows the data that are available to analyze. To more usefully illustrate how to apply a user-defined statistical test of interest, we construct here a toy example, the “show2” function, which computes Fisher’s exact test for the trait vs marker and shows the p-value.

show2 = function(m, r, e) {
   fn = function(x) {
          tryCatch(fisher.test(table(e$fam$trait, x)),
                   error = function(e) {list(p.value = NA)}) $p.value }
      print(r)
# collect genotypes for the set of markers "m"
      mm = getgenotypes(m, envir=e)
# apply fisher.test of trait vs marker for each marker
      pv = apply(mm, 2, fn)
      names(pv) = m$MarkerName
      print(pv)
}

Now we can use the *applyFnToGenes* function to apply Fisher’s exact test to each marker within the CEP104 gene.

applyFnToGenes(show2, genes_arg = c("CEP104"))

## ’select()’ returned 1:1 mapping between keys and columns
## ’select()’ returned 1:many mapping between keys and columns

##   ENTREZID   ALIAS  SYMBOL TXID       TXNAME TXCHROM TXSTRAND TXSTART   TXEND
## 1     9731  CEP104  CEP104 4281   uc001aky.2       1        - 3728645 3773797
##  snp24037   snp24039   snp24041    snp24048  snp24494  snp24499  snp24506
## 0.2649470  0.9628246  1.0000000   0.2059075 0.6286297 0.6897829 0.6897829
##  snp24507
## 0.6286297

Note: Since Fisher’s test is a function of unique categorical values, the *getgenotypes* function in “show2” could be replaced by *getgenotypesraw* without changing the calculated p-values.

### Using Mega2R to carry out automated gene-based association tests using ’pedgene’

Mega2R provides functions that permit one to run the ’pedgene’ function by Schaid *et al.* ^18^ to carry out gene-based association tests on family data looping over selected transcripts.

### Loading a Mega2 database with *init_pedgene*

Rather than read the Mega2 SQLite database with the *read.Mega2DB* function as described previously, here we use a specialized *init_pedgene* function to read the Mega2 database. This latter function calls a utility function also used by *read.Mega2DB*. Then it creates, edits, and rewrites the family (.fam) data, storing it in a ’pedgene’-compatible data frame, **fam.** ( **fam** merges data from the **pedigree_table**, **person_table** and **phenotype_table**.) (When **fam** is updated, filtering is automatically done to the persons in the **phenotype_table** and the **unified_genotype_table** to remove any persons removed from **fam.**) Finally, *init_pedgene* calculates some values that will be used repeatedly and stores them in the environment that is returned.

ENV = init_pedgene("seqsimr.db")

### Gene ranges Reminder

Mega2R has an internal default list of the chromosome and base pair ranges for a number of gene transcripts. These transcripts come from the UCSC Genome Browser reference assembly GRCH37. The list was further modified to eliminate multiple records of the same gene with the exact same transcript start and transcript end. These data contain about 29,000 records. You may use the *setRanges* function to load your own range set instead of the default, as described above.

### Gene transcripts reminder

By default, the *init_pedgene* function sets the transcript database to “TxDb.Hsapiens.UCSC.hg19.knownGene” and the Entrez gene name mapping database to “org.Hs.eg.db”. If you wish to use different databases to select transcripts by gene name, you must use the *setAnnotations* function to load them from Bioconductor, as discussed above.

### Running pedgene on ranges

By default, the function *Mega2pedgene* examines the first 100 transcripts and prints the results. (Internally, it calls the function *applyFnToRanges*.) For the seqsimr.db database, the first 100 transcripts contain only one transcript with several markers. To make this Use Case run faster, we noticed that the identified transcript appeared at transcript 54; so we will restrict *Mega2pedgene* to a small range of transcripts around 54, viz. 53 through 55.

Note: ’verbose’ needs to be TRUE, for the diagnostics to be printed.

ENV$verbose=TRUE
Mega2pedgene(gs=53:55)

## tryFn() No markers in range:  NM_014705, DOCK4, 7, 111366163, 111846462
## tryFn() No markers in range:  NM_003759, SLC4A4, 4, 72204769, 72437804

## CEP104 snp24037 520 646 214
## CEP104 snp24039 940 386 54
## CEP104 snp24041 1377 3 0
## CEP104 snp24048 860 460 60
## CEP104 snp24494 789 501 90
## CEP104 snp24499 891 442 47
## CEP104 snp24506 891 442 47
## CEP104 snp24507 789 501 90
##   chr   gene nvariants   start     end sKernel_BT pKernel_BT sBurden_BT
## 1   1 CEP104         8 3728644 3773797   31.96998  0.6297541 -0.6496837
##   pBurden_BT sKernel_MB pKernel_MB sBurden_MB pBurden_MB sKernel_UW
## 1  0.5158965   1184.995  0.5138866  -1.209563  0.2264468   222.8737
##   pKernel_UW sBurden_UW pBurden_UW
## 1  0.4111136  -1.169488   0.242207

You will see some reports of “No markers in range”, because the database only contains markers on a sub range of chromosome 1 whereas the transcripts span the entire genome. Then you will see a listing of a gene name, a marker name, and count of the 1/1, 1/2, and 2/2 genotypes (where ’1’ is the major allele), e.g.,.

         CEP104 snp24037 520 646 214
        ...

The markers, the range used, and the environment are passed to the callback function *DOpedgene*. *DOpedgene* converts the raw genotype representation returned by *getgenotypesraw* to the values 0, 1 and 2. Then it runs ’pedgene’. The results are automatically stored in a data frame with columns: chromosome, gene, number of markers and base pair range followed by ’pedgene’ data: kernel and burden, value and p-values, four values for each of three weightings of the markers. These data are saved in the data frame, ’pedgene_results’, in the environment. They are also printed when ’verbose’ is TRUE.

Note: The results are always appended to the ’pedgene_results’ data frame. You should truncate it when necessary.

You could run *Mega2pedgene* on all the transcript entries, but it takes a rather long time. You would type:

Mega2pedgene(gs=1:nrow(ENV$refRanges), envir = ENV)

If you run the above test, you will see that genes DISP1 and KIF26B have at least one p-value less than 0.01 and AK5 and STL7 at least one less than 0.03.

### Running pedgene on selected genes

You may try searching for transcripts of specific genes. Here, the default transcript database is "TxDb.Hsapiens.UCSC.hg19.knownGene" from Bioconductor. Of course you can change it, if you install a new database from Bioconductor, as shown earlier.

We leave the command below as an exercise, as it runs a bit slowly. It needs to find all the transcripts for each gene, then to find all the markers between each pair of transcript start/end ranges, then to compute the genotype matrix for these markers, and finally to call the callback function with the appropriate arguments.

applyFnToGenes(DOpedgene, genes_arg = c('DISP1', 'KIF26B', 'AK5', 'ST7L'), envir = ENV)

But let us run this function for a few genes:

# turn off output & reset pedgene_results data frame
ENV$verbose = FALSE
ENV$pedgene_results = ENV$pedgene_results[0, ]

applyFnToGenes(DOpedgene, genes_arg = c('DISP1', 'AK5'), envir = ENV)

## ’select()’ returned 1:1 mapping between keys and columns
## ’select()’ returned 1:many mapping between keys and columns

print(ENV$pedgene_results)

##   chr  gene nvariants     start       end sKernel_BT  pKernel_BT
## 1   1   AK5         4  77747662  78025654   2593.945 0.228311038
## 2   1 DISP1         4 222988431 223179337   4559.340 0.004224181
##   sBurden_BT pBurden_BT sKernel_MB  pKernel_MB sBurden_MB  pBurden_MB
## 1  -1.400928  0.1612355   1899.249 0.062069786  -2.263002 0.023635537
## 2   1.446520  0.1480315   5410.868 0.000253584   2.916462 0.003540261
##   sKernel_UW pKernel_UW sBurden_UW pBurden_UW
## 1   175.4195 0.05101300  -2.290307 0.02200350
## 2   277.5080 0.04407319   2.094983 0.03617254

## Summary

As with all software projects, **Mega2R** is a work in progress, and so there are a number of possible improvements that could be made in the future. These include:

• defer constructing the **unified_genotype_table** until the set of chromosomes that are needed can be computed and then only read the required chromosome genotype vectors.
• analogously to Mega2SKAT, add support for the R famSKATRC package ^30, 31^. famSKAT_RC is another package for family or unrelated gene-based association analysis for a mix of rare variants and common variants.
• analogously to Mega2GenABEL, add support for the CoreArray data storage system ( *e.g.*, Genomic Data Structure (GDS) format ^32^). This will support conversion to GDS format in both the SNPRelate and the SeqArray data representations ^15^.
• apply more aggressive compression of the database. For one, the genotype raw vector can be further compressed by gzip or a similar compression protocol. Additionally, since C does not have a way to represent missing data, we use -99.99 to represent it which is stored in the database as a floating point number (8 bytes). But the database (via NULL) and R (via NA) can efficiently represent a missing values, so we will will convert C missing (-99.99) to SQLite (NULL).

## Data availability

All data underlying the results are available as part of the article and no additional source data are required.

## Software availability

Software name: **Mega2R**

Software available from: https://CRAN.R-project.org/package=Mega2R

BitBucket repository: https://bitbucket.org/dweeks/mega2r/

Documentation page: https://watson.hgen.pitt.edu/register/docs/mega2R.html

Operating system(s): Platform independent

Programming language: R, C++

Other requirements; R, SQLite library

Archived source code (v1.0.4) at the time of publication: https://doi.org/10.5281/zenodo.1343587 ^33^

License: GNU GPL 2

## Supplementary material

Supplementary File 1: The Mega2R package: R tools for accessing and processing genetic data in common formats. PDF contains detailed descriptions of the data frames that Mega2R makes available.
