# Supplementary material for: The Mega2R package: R tools for accessing and processing genetic data in common formats
Source: F1000Res. 2019 Feb 25;7:1352. Originally published 2018 Aug 29. [Version 2] doi: 10.12688/f1000research.15949.2 (PMC6137409; doi:10.12688/f1000research.15949.2)
Supplement: Supplementary file 1 [file f1000research-7-19809-s0000.tgz › a6ace9cb-cb34-4e6c-91c6-651c480d5e67_Mega2R_Supplementary_Material_v2.pdf]

Supplementary Material

The Mega2R package: R tools for accessing and processing genetic data in common formats

Robert V. Baron[1], Justin R. Stickel[1], and Daniel E. Weeks[1,2,*]

[1]Department of Human Genetics, Graduate School of Public Health, University of Pittsburgh, Pittsburgh, Pennsylvania, 15261, USA
[2]Department of Biostatistics, Graduate School of Public Health, University of Pittsburgh, Pittsburgh, Pennsylvania, 15261, USA
[*]Corresponding author
Email: Robert V. Baron - rvb5@pitt.edu, Justin R. Stickel - jrs110@pitt.edu, Daniel E. Weeks - weeks@pitt.edu

Supplementary Material

The data from most of the Mega2 SQLite tables are potentially useful and are made available in data frames.

Miscellaneous Tables

int_table

This table contains data like Phenotype Count and Locus Count; they can be calculated from tables but its faster to have the value available. There also are a **double_table**, **charstar_table** and **stuff_table** for storing: double float, character string and byte vector values, respectively.

column	contents
pId	unique database table key
key	name of an integer
value	value for integer

Pedigree & Person Tables

pedigree_table (and pedigree_brkloop_table)

This table provides the basic data for a family: name, an index and count of members.

column	contents
pId	unique database table key
Num	unique ID for pedigree
EntryCnt	number of persons in pedigree
Name	Mega2 alternate name
PedPre	ID from study
OriginalID	Mega2 alternate name
origped	Mega2 alternate name
pedigree_link	unique integer linking family info

person_table (and person_brkloop_table)

This table provides the basic data for a person: name, an index, parents, and sex.

column	contents
pId	unique database table key
UniqueID	unique ID for person
OrigID	Mega2 alternate name
FamName	Mega2 alternate family name
PerPre	ID from study
ID	numeric ID for person
Father	father's ID
Mother	mother's ID
Sex	person's sex
pedigree_link	unique integer linking family info
person_link	unique integer linking person info

pedigree_brkloop_table and person_brkloop_table

These tables are available for the analyses that do not accept pedigrees with loops. The loops are broken and these tables create a "doppelgänger" for one person in each loop and adjust the parents of one of the pair to break the loop. Compared to the pedigree_table and person_table, there will be an extra person in some families (those with loops).

Marker Information Tables

locus_table

This table provides the basic data for a locus: its name, type and allele count.

column	contents
pId	unique database table key
LocusName	locus (phenotype) name
Type	QUANT, AFFECTION, BINARY, NUMBERED, XLINKED, YLINKED
AlleleCnt	number of alleles for this marker
locus_link	unique integer linking loci info

allele_table

This table list the allele data for each locus: name, frequency and a index increasing monotonically within the locus.

column	contents
pId	unique database table key
AlleleName	allele nucleotide
Frequency	allele frequency
indexX	numeric index of allele 1-N
locus_link	unique integer linking loci info

marker_table

This table provides the basic data for a marker: its name, chromosome and position.

column	contents
pId	unique database table key
MarkerName	name of marker
pos_avg	preferred average genetic position
pos_female	preferred female genetic position
pos_male	preferred male genetic position
chromosome	chromosome of marker
locus_link	unique integer linking loci info
locus_link_fill	unique integer indexing into the unified genotype table

markerscheme_table

This table is used as part of the compression algorithm to compress nucleotide values.

column	contents
pId	unique database table key
key	unique integer linking loci
allele1	allele 1 value
allele2	allele 2 value

map_table

This table provides the genetic position values for a given marker according to the “map” index defined in the table below.

column	contents
pId	unique database table key
marker	unique integer linking loci
map	map index number
position	average genetic position
pos_female	female genetic position
pos_male	male genetic position

mapnames_table

This table provides a mapping of a map name string to a map index. If all of *sex_averaged_map*, *male_sex_map* and *female_sex_map* are 0, the corresponding map's *position* specifies a base pair position.

column	contents
pId	unique database table key
map	map index number
sex_averaged_map	1 if present else 0
male_sex_map	1 if present else 0
female_sex_map	1 if present else 0
name	map name

Phenotype Tables

traitaff_table

This table indicates the number of classes and number of penetrance entries for each affection locus.

column	contents
pId	unique database table key
ClassCnt	number of liability classes
PenCnt	number of penetrance values
locus_link	unique integer linking loci info

affectclass_table

This table holds penetrance data for each loci for each class.

column	contents
pId	unique database table key
MaleDef	0 indicates corresponding penetrance was set
FemaleDef	0 indicates corresponding penetrance was set
AutoDef	0 indicates corresponding penetrance was set
MalePen	male penetrance
FemalePen	female penetrance
AutoPen	autosomal penetrance
locus_link	unique integer linking loci info
class_link	unique integer specifying class index

phenotype_table

This table holds phenotype data for each person.

column	contents
pId	unique database table key
person_link	unique integer linking person info
bytes	byte count of data
data	raw data

Unified Genotype Table

unified_genotype_table

This table holds genotype data for each person.

column	contents
person_link	unique integer linking person info
data	raw data
