# Supplementary material for: The Mega2R package: R tools for accessing and processing genetic data in common formats
Source: F1000Res. 2019 Feb 25;7:1352. Originally published 2018 Aug 29. [Version 2] doi: 10.12688/f1000research.15949.2 (PMC6137409; doi:10.12688/f1000research.15949.2)
Supplement: Peer review history

# Peer review: Reviewer response for version 2

**DOI:** 10.5256/f1000research.19809.r44919

**Published:** 2019-02-25

**Authors:** Derek Gordon

**Affiliations:** 1 Department of Genetics, Rutgers, The State University of New Jersey, Piscataway, NJ, USA

No changes required.

I have read this submission. I believe that I have an appropriate level of expertise to confirm that it is of an acceptable scientific standard.

# Peer review: Reviewer response for version 2

**DOI:** 10.5256/f1000research.19809.r44920

**Published:** 2019-02-25

**Authors:** Alejandro Q. Nato, Jr.

**Affiliations:** 1 Department of Biomedical Sciences, Joan C. Edwards School of Medicine, Marshall University, Huntington, WV, USA

The authors have adequately responded to all of the reviewers' comments. I do not have any further comments to make.

I have read this submission. I believe that I have an appropriate level of expertise to confirm that it is of an acceptable scientific standard.

# Peer review: Reviewer response for version 1

**DOI:** 10.5256/f1000research.17420.r37773

**Published:** 2018-10-09

**Authors:** Derek Gordon

**Affiliations:** 1 Department of Genetics, Rutgers, The State University of New Jersey, Piscataway, NJ, USA

For those who perform computational analysis in the fields of statistical genetics, bioinformatics, or population genetics, it is no exaggeration to say that the lion’s share of one’s time is spent formatting data so that it can be input into a specific analysis program. Having worked in statistical genetics for over two decades, this reviewer recalls a time when commonly used linkage programs such as LINKAGE, GENEHUNTER, S.A.G.E., LIPED, SIMWALK2, and others, each had different data input formats. For those format conversion programs that existed, they worked for one or a small number of other programs (e.g., conversion from LINKAGE format to S.A.G.E. format). Also, the programs were usually lab-specific, in that they were not publically available, or if they were, there was no “accountability”; that is, there was no error checking for the code, nor was there a way to send “bug reports” to the programmer.

 That all changed when the first version of Mega2 was released. It was the “Rosetta Stone” for researchers who performed computational analyses. Not only did it allow for one to convert from one format to another seamlessly, it performed data error checking, created log files with each run of the program, and critically, it created Unix/Linux shell scripts that greatly simplified analysis runs.

 The importance of data storage and format conversion has only grown over time. Like the adage states, the only constant in life is change. The genomic data employed for linkage, association, and other computational analyses has grown in complexity and storage requirements. A list of genomic data employed in computational studies, from an approximate chronological standpoint, contains: di-allelic RFLPs, multi-allelic VNTRs, di-allelic SNPs, quantitative copy number variants (CNVs), next generation sequence, and RNA-seq data. As an example of the complexity of statistical methods to analyze these data, a PubMed search using the key terms “next generation sequencing”, “statistical method” and “software” yields over 350 hits.

 The practice of program-specific formatting continues. Programs like PLINK, MACH, and METAL have their own input formats. While some of the input files (like the pedigree files) may be similar, other files like those that contain chromosome and base-pair position for a given item (e.g., SNP), may be different.

 Given the number of statistical methods being employed, that they will most likely have different formats, that error checking is critical, and that data storage for the volumes of data now being generated must be efficient, it is critical that Mega2R be recognized. Mega2 has enjoyed a long-standing tradition as the “gold-standard” program for data format conversion. Because  Dr. Weeks has directed the updating and stability of Mega2 in its various versions, and continues to do so with Mega2R, one can reasonably expect that Mega2R will be widely used, widely cited, and most especially will make the life of computational geneticists much easier.

I have read this submission. I believe that I have an appropriate level of expertise to confirm that it is of an acceptable scientific standard.

# Peer review: Reviewer response for version 1

**DOI:** 10.5256/f1000research.17420.r37770

**Published:** 2018-09-13

**Authors:** Alejandro Q. Nato, Jr.

**Affiliations:** 1 Department of Biomedical Sciences, Joan C. Edwards School of Medicine, Marshall University, Huntington, WV, USA

Baron, Stickel, and Weeks present a well-written article describing the newly-developed R package (Mega2R) as an additional tool to the robust Mega2 data-reformatting software. Mega2R helps alleviate problems in handling huge genetic data by allowing the user to specify regions of the genome, genes, or markers that will be included in the analysis. It keeps the genotype data in a compressed format when reading from the Mega2 SQLite database to efficiently overcome the memory restrictions of R. It includes several functions that allow a user to carry out additional association tests, outputs Mega2R data as a VCF file with other related files, converts data frames into GenABEL-compatible format, and enhances GenABEL by supporting additional input data formats. Mega2R is indeed a useful R package for genetic analysis.

 I enjoyed reading the manuscript since the authors have clearly described what a user can do through Mega2R.

 I have a few minor comments and suggestions below:

1. Add a schematic diagram of Mega2R showing the different functions and how they interact with the SQLite database. This will visually help readers understand this article.
2. For future enhancements, would it be possible to have a *Mega2pedgene* function that allows the user to choose between using reference/alternate alleles instead of major/minor alleles? This may allow Mega2R to facilitate analysis involving datasets from different populations.
3. Is the chunk of 1,000 markers at a time in building the VCF file a fixed value? Or will you allow the user to select a particular number in the future?
4. Indicate the format of the genotype matrix, e.g., normal format (markers in columns and individuals in rows), based on your examples.
5. Will Mega2R be back compatible or will it only be compatible with R 3.5.0 onwards?
6. Typographical error (retruns)

I have read this submission. I believe that I have an appropriate level of expertise to confirm that it is of an acceptable scientific standard.

# Response

**Published:** 2019-01-25

**Authors:** Daniel Weeks

**Affiliations:** Department of Human Genetics, University of Pittsburgh, USA

Thank you for your thoughtful comments and suggestions for strengthening our paper.

In Version 2, as suggested, we have added two Figures, in the 'Implementation' section, which now provide graphically illustrate how Mega2 and Mega2R work together (Figure 1) and how Mega2R provides an efficient and flexible wrapper for iterating through gene regions (Figure 2).  However, this does not directly address your request for a diagram showing how the different Mega2R functions interact with the SQLite database.  We did not add such a diagram because the interaction is minimal – at the beginning, we load the SQLite Mega2 databases directly into R as data frames for further analysis and manipulation.  After this step, the functions use the loaded data frames.

 Regarding your numbered comments, we respond here:

1. Using reference/alternate alleles within Mega2R instead of major/minor alleles is not yet possible, but this would definitely be a useful future enhancement.

2. Currently Mega2R's chunk size is defined by an internal parameter, so altering the chunk size could be accomplished by altering the source code.  Making this a parameter would also be a useful future enhancement.

3. The genotype matrix has individuals in rows and markers in columns. This was indicated in the paragraph that first introduces the getgenotypes and getgenotypesraw functions: "The functions getgenotypes and getgenotypesraw return a matrix of nucleotide pairs or a matrix of encoded integers with a column for each marker and containing a row per sample."  As suggested, in this revision we have added a comment to the 'show' function clarifying this later in the manuscript.

4. Due to constraints encountered when submitted the Mega2R package to CRAN, Mega2R requires an R version of 3.5.0 or higher.  As version 3.5.0 was released some time ago, in April 2018, hopefully this version requirement is not a burden.

5. The typographical error (retruns) has been corrected.

# Peer review: Reviewer response for version 1

**DOI:** 10.5256/f1000research.17420.r37771

**Published:** 2018-09-11

**Authors:** Dajiang J. Lui

**Affiliations:** 1 Department of Public Health Sciences, Institute of Personalized Medicine, Penn State College of Medicine, Hershey, PA, USA

Weeks and colleagues described a very useful R package to access multiple data formats and facilitate its integration with R packages. This package is based upon its predecessor MEGA2, which is highly cited and used in genetic data analysis. 

 This tools is long overdue. While numerous statistical genetics methods are being developed, only a minority of them have really a usable interface. Due to the metadata that modern genetic datasets needs= to store, many of the files are not stored in a rectangular format. Parsing these files and making them analyzable in R is often the most time-consuming part to code but is the key for the development. The package filled in this gap and make easy the development of useful softwares. 

 I only have a few minor comments: 

 1) The software is unique and useful, but the existence of other packages that can read and parse genetic datasets in R should be acknowledged. For example, VariantAnnotation package in bioconductor and seqminer in CRAN can both randomly access tabix indexed VCF/BCF files. 

 2) One of the major strength of the package is to facilitate statistical genetics method development. So it would be helpful if the authors could give a toy example on how to make use of applyFnToGenes to handle a user defined statistical tests.

I have read this submission. I believe that I have an appropriate level of expertise to confirm that it is of an acceptable scientific standard.

# Response

**Published:** 2019-01-25

**Authors:** Daniel Weeks

**Affiliations:** Department of Human Genetics, University of Pittsburgh, USA

Thank you for your helpful suggestions for improving our paper.

 In Version 2, as suggested, we now cite, in the Introduction, other R and Bioconductor packages that can read and parse genetic datasets.

 As suggested, we have added, to the Use Case section, a toy example illustrating how to use applyFnToGenes to apply a user-defined statistical test.
